# Incidence of incisional hernia in patients undergoing cytoreductive surgery and hyperthermic intraperitoneal chemotherapy: an observational clinical study from a tertiary oncology referral care center in India

**DOI:** 10.1186/s12957-024-03408-3

**Published:** 2024-05-17

**Authors:** Mukurdipi Ray, Amit Kumar, Haraesh Maranna

**Affiliations:** Department of Surgical Oncology, Dr BRA IRCH, AIIMS, New Delhi, 110029 India

**Keywords:** CRS, HIPEC, Incisional hernia, Peritoneal surface malignancy

## Abstract

**Background:**

An incisional hernia (IH) after major abdominal surgery is an unwanted complication particularly following cytoreductive surgery and hyperthermic intraperitoneal chemotherapy (CRS and HIPEC). The frequency of IH among patients treated with CRS and HIPEC remains unexpectedly high in various studies. This study aimed to analyze the incidence, determine the factors contributing to the occurrence of IH, and develop methods to reduce the incidence of IH.

**Methods:**

We retrospectively analyzed data from a prospectively maintained structured computerized comprehensive database of 360 patients who had undergone CRS and HIPEC after January 2013 and completed two years of follow-up before December 2023. All patients were followed for a minimum period of two years with physical examination and radiological imaging when required and the occurrence of IH was documented. We used SPSS software version 24 to analyze the data using appropriate statistical tests. We set a significance threshold of *p* < 0.05.

**Results:**

Within two years of undergoing CRS and HIPEC, 25 patients (6.9%) out of 360 developed IH, indicating an annual incidence rate of 3.5%. The mean duration of hospitalization for the CRS/HIPEC procedure was 8.4 ± 4.13 days. Fifty-two (14.4%) patients experienced early post-operative surgical complications. The development of IH in our series was significantly associated with obesity (76% vs. 8.4%, *P* = 0.001), the occurrence of early post-operative surgical complications (48% vs. 12%, *P* = 0.001), mainly category III complications (44% vs. 7.1%), category IV complications (24% vs. 2.9%) according to Clavien-Dindo classification, post neoadjuvant chemotherapy status (72% vs. 87%, *P* = 0.045) and need for bowel anastomosis (32% vs. 11%, *P* = 0.002).

**Conclusion:**

The lower incidence of IH following CRS and HIPEC in our patient cohort than in the literature can be attributed to a combination of factors, including the use of meticulous surgical techniques and the use of an abdominal binder postoperatively, particularly in obese patients.

## Background

An incisional hernia (IH) may develop in 11–20% of patients, during a follow-up period that usually lasts for 12 to 20 months after midline laparotomy [1–4]. Various factors may increase the risk of developing IH after laparotomies, which may be related to patient or treatment characteristics. These potential risk factors include obesity, prior surgery, ascites, female sex, a low ratio of suture length to wound length, infections specific to the surgical site, prolonged surgery duration, longer incisions, and the use of interrupted sutures [5–7]. A study conducted at a single center showed that 43% of the 491 patients who had undergone surgery to remove intra-abdominal cancer experienced IH [8]. While the majority of IH patients do not manifest any symptoms, the presence of IH can result in significant discomfort and pain, as well as reduced physical abilities and a negative impact on one’s body image [9, 10]. In some instances, IH may lead to obstruction and strangulation, necessitating urgent surgical intervention. Additionally, the rate of long-term IH recurrence following repair is approximately 30% [11]. Hence it is vital to anticipate and prevent IH to improve patients’ quality of life.

The management of peritoneal metastases arising from abdominal tumors such as appendiceal neoplasms, colorectal carcinoma, ovarian malignancies, and mesotheliomas of the peritoneum is increasingly being carried out through a combination of CRS and HIPEC. CRS involves midline laparotomy with an incision that extends from the xiphoid to the pubic symphysis [12, 13]. The completion of cytoreduction (CC) score is used to define optimal and suboptimal CRS and ranges from 0 to 3 [14, 15]. Although CRS and HIPEC are linked to significant post-operative complications [16], studies have shown their association with favorable oncologic outcomes in the long term [17]. Patients who undergo CRS and HIPEC, surgical procedures involving the delivery of intraperitoneal chemotherapy, are at risk of developing hernias due to several factors. These include previous abdominal surgeries, post neoadjuvant chemotherapy, nutritional status, immunosuppressed state, the length of the procedure, long incisions, the presence of ascites, and the localized effect caused by peritonectomy and intraperitoneal chemotherapy [18, 19].

Based on an experimental trial, Boutros et al. were the first to suggest that CRS and HIPEC could increase the risk of IH. The reason being that during CRS, a lot of tumor tissue is removed from the peritoneal cavity and further HIPEC involves infusion of heated chemotherapeutic drugs into the abdomen. These procedures weaken up the abdominal muscles, allowing the abdominal contents to protrude through the surgical incision site. Moreover, there is extensive tissue trauma, manipulation and impaired bone healing. Even the heat from HIPEC contributes to this tissue integrity and delayed wound healing, predisposing the surgical site to hernias. However, there has been limited research on the frequency of IH following CRS and HIPEC, and the incidence rates reported in these studies have varied greatly, ranging from 9.2% [20] to 26.9% [21].

This study aimed to determine the factors contributing to the occurrence of IH within two years following CRS and HIPEC and to devise methods to reduce the incidence of IH, thereby preventing unnecessary surgical interventions.

## Methods

We retrospectively analyzed the prospectively maintained structured computerized comprehensive peritoneal surface malignancy database of 360 patients who underwent CRS and HIPEC in the Department of Surgical Oncology of a high-volume tertiary care cancer center, BRA IRCH AIIMS NEW DELHI, INDIA. The primary objective of this study was to establish the incidence of IH, defined as a gap in the abdominal wall, which may or may not result in a protrusion in the region of a surgical incision identified on physical examination or radiological imaging. The secondary objective was to assess risk factors for IH after CRS and HIPEC. Inguinal hernia and parastomal hernia were not assessed as a part of study.

### Inclusion and exclusion criteria

This study included all patients who had undergone CRS and HIPEC after January 2013 and completed a minimum follow-up of two years before December 2023. Patients who had undergone laparoscopy only, prior mesh reconstruction for abdominal wall resection, recurrent cases of abdominal malignancy, inadequate data, unavailable radiological images, and patients who were lost to follow-up were excluded from the study.

### Surgical techniques

**Fig. 1:**
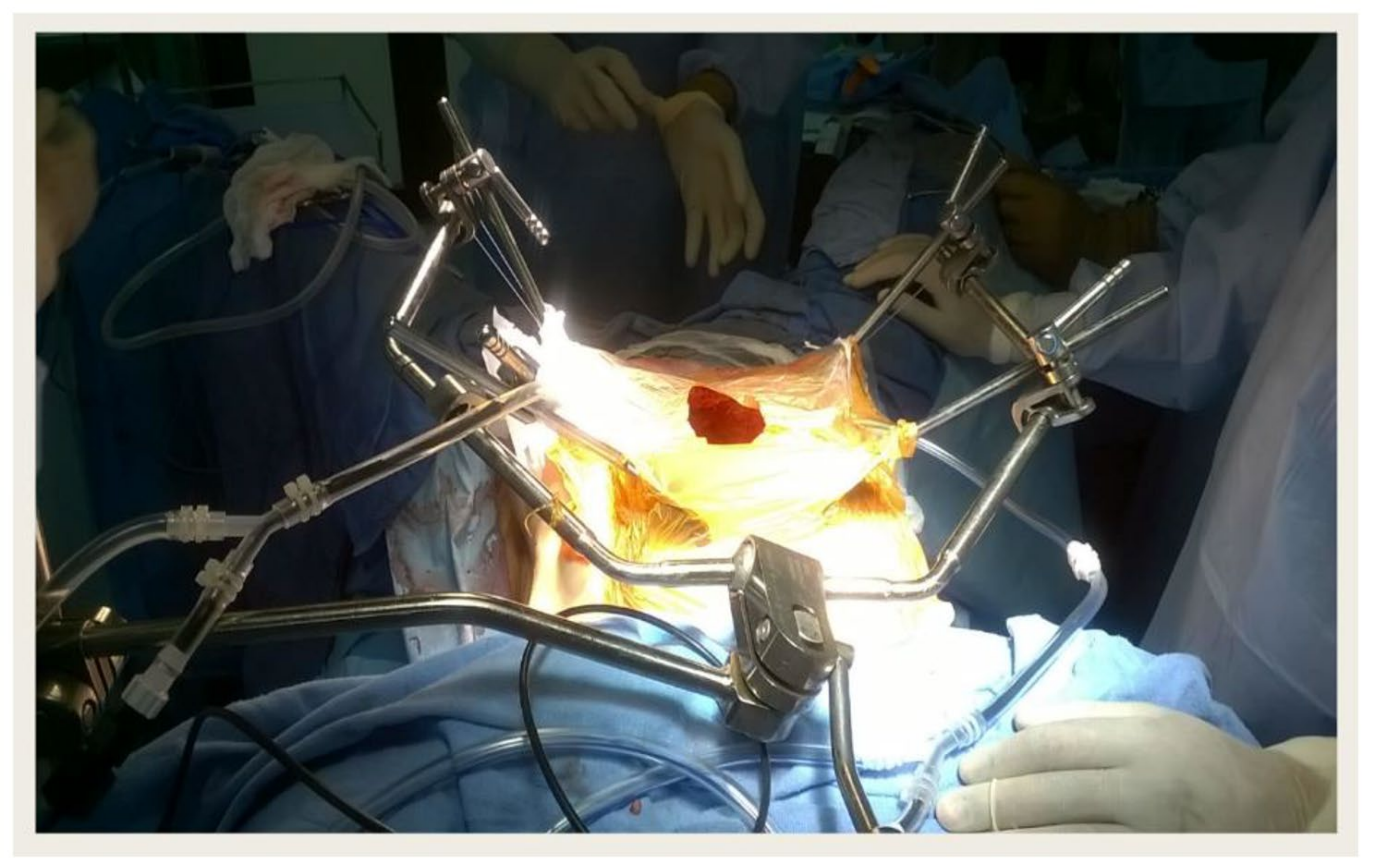
Semi -open HIPEC technique

A midline incision was made from the xiphisternum to the pubic symphysis to commence CRS and HIPEC [20]. A primary survey was performed to evaluate the peritoneal carcinomatosis index (PCI) [22]. Complete cytoreduction was performed to remove all visible diseases. A disease-specific or total parietal peritonectomy, total omentectomy, removal of primary tumor, pelvic and/or retroperitoneal lymphadenectomy along with visceral resection based on the disease extent were performed. The completeness of the cytoreduction (CC) score was estimated after the completion of the procedure [23]. CRS was followed by HIPEC perfusion for patients who had optimal CRS (CC 0,1). All bowel anastomosis was performed before HIPEC either hand-sewn or stapled. Intraperitoneal chemotherapy was administered via a semi-open technique with a temperature set at 41–43˚C for 60 to 90 min. The most commonly used drug for HIPEC was cisplatin (75–80 mg/m^2^) followed by mitomycin C (15–20 mg/m^2^) and doxorubicin (15–20 mg/m2). The midline incision was closed using a monofilament slowly absorbable loop polydioxanone suture (PDS) (2 − 0). We used two PDS sutures starting at both ends of the midline incision via a continuous suturing technique and finally tied the knot on one side of the midline. We used the old golden “Rule of 1”, i.e. a distance of 1 cm from the margin, a depth of 1 cm, and a distance of 1 cm from each suture site. Skin closure was performed with interrupted polyamide sutures (2 − 0). We place either one or two intra-abdominal drains and remove them between the 3rd to 5th post-operative days. In obese patients, a cavity drain was placed in the subcutaneous plane and removed when the output was < 10 ml for three consecutive days. We used an abdominal binder to prevent lateral abdominal wall tension postoperatively in all patients. Perioperative intravenous antibiotic prophylaxis (Sulbactam and Cefoperazone combination 1gm twice daily) was administered for three days and post-operative complications were classified using the Clavien-Dindo classification [24]. Figure 1 shows the representative photo of the surgical technique.

### Data collection

Patients were subjected to regular physical examinations every 2 months after discharge as per institutional protocol. Typically, abdominal ultrasound was conducted every 3 months for a duration of 2 years, and computed tomography (CT scans) were taken at 6-month intervals in the high-risk group, (e.g. obese and those with multiple abdominal surgeries), otherwise yearly, to detect disease recurrence. Patients were followed for a minimum of 2 years. The data collected also included demographic details, clinical presentation, treatment details, staging, and post-operative complications.

### Statistical analysis

To analyze the data, we utilized the SPSS software package version 24. To represent the numerical and proportional data, we used categorical variables, while the continuous variables are expressed as the means with standard deviations. To determine the correlation between groups, we employed a chi-squared test or Fisher’s exact test for categorical variables and a Mann-Whitney U test or Student’s t-test for continuous variables. To establish statistical significance, we set a *p*-value threshold of less than 0.05.

## Results

We enrolled 360 eligible patients who underwent CRS and HIPEC at a single center. The study included 313 (86.9%) females and 47 (13.1%) males, with an average age of 48 ± 11.53 years. Table 1 describes the baseline characteristics of the study population.

**Table 1:** Baseline characteristics of the study population

Characteristics	*N* = 360
**Mean Age ± std. deviation (years)**	48.0 ± 11.53
**Sex**	
Female	313 (86.9%)
Male	47 (13.1%)
**Previous abdominal surgeries**	52 (14.4%)
**Comorbidities**	
Hypertension	29 (8.1%)
Diabetes	22 (6.1%)
Hypothyroidism	21 (5.8%)
Obesity (BMI > 30 Kg/m^2^)	47 (13.1%)
**Diagnosis/Primary malignancy**	
Ovarian carcinoma	231 (64.2%)
Colorectal Carcinoma	39 (10.8%)
Pseudomyxoma peritonei	36 (10%)
Appendiceal neoplasm	22 (6.1%)
Uterine sarcoma	10 (2.8%)
Gastric carcinoma	10 (2.8%)
Peritoneal mesothelioma	10 (2.8%)
Small bowel carcinoma	2 (0.6%)

The mean duration of hospitalization was 8.4 ± 4.13 days. Among the 360 patients, 52 (14.4%) had early post-operative surgical complications. The frequencies of different post-operative complications are given in Table 2. Two patients developed post-operative anemia significant enough to need transfusion, but re-exploration was not required in either of the cases.

**Table 2:** Summary of early post-operative surgical complications

Early Post-operative Surgical complications	Frequency	Percentage (*n* = 360)
Anastomotic leak	6	1.67%
Bile Leak	2	0.56%
Bleeding (requiring blood transfusion)	2	0.56%
Burst abdomen	6	1.67%
Surgical site infection	19	5.27%
Enterocutaneous fistula	5	1.39%
Intra-abdominal collection	3	0.83%
Bowel perforation	3	0.83%
Subacute intestinal obstruction	6	1.67%
**Clavien-Dindo classification**		
0	238	66.11%
I	40	11.11%
II	27	7.50
III	35	9.72%
IV	16	4.44%
V	4	1.11%

**Fig. 2:**
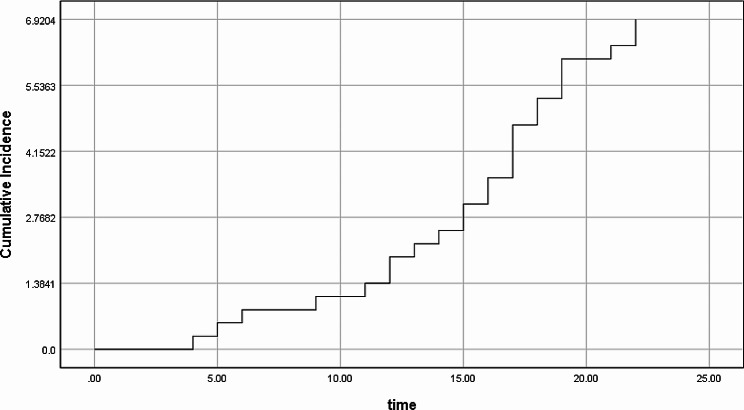
Kaplan-Meier curve for cumulative incidence proportion of incisional hernias after CRS-HIPEC

Within two years of CRS/HIPEC, 25 (6.9%) patients developed IH leading to an annual incidence of 3.5%. The median time to occurrence of IH after surgery was 16 months and the cumulative incidence proportion of IH is presented graphically in Fig. 2.

Obesity, early post-operative surgical complications (48% vs. 12%, *P* = 0.001), mainly category III (44% vs. 7.1%) and category IV (24% vs. 2.9%) Clavien-Dindo classification, need for bowel anastomosis during the CRS/HIPEC procedure (*P* = 0.002), and post-NACT (neo-adjuvant chemotherapy) status (*P* = 0.045), were significantly associated with the development of IH within 2 years of surgery, as shown in Table 3. On multivariate analysis too, obesity, early post-operative surgical complications, Clavien-Dindo grade of complications and post-NACT status were observed as independent risk factors for development of IH as described in Table 4.

**Table 3:** Factors associated with incisional hernia: univariate analysis

Characteristics	Patients with incisional hernia (*n* = 25)	Patients without incisional hernia (*n* = 335)	*P*- value
**Mean Age ± std. deviation (years)**	48.1 + 11.53	47.0 ± 8.05	0.642
**Sex**			
Female	21	292	0.551
Male	4	43	
**Previous abdominal surgeries**			
None	18	290	0.87
Yes	7	45	
**Comorbidities**			
Hypertension	2	27	0.68
Diabetes	0	22	0.384
Hypothyroidism	0	21	0.382
Obesity	19	28	**0.001**
**Diagnosis/Primary malignancy**			
Ovarian carcinoma	18	213	0.77
Colorectal carcinoma	3	36	
Pseudomyxoma peritonei	1	35	
Appendiceal neoplasm	1	21	
Uterine sarcoma	0	10	
Gastric carcinoma	0	10	
Peritoneal mesothelioma	2	8	
Small bowel carcinoma	0	2	
**Post NACT status**			
Yes	18	290	0.045*
No	7	45	
**PCI score**			
< 15 (low)	16	184	0.227
*≥* 15 (high)	5	47	0.778
**Intraoperative details**			
Mean duration of surgery (minutes)	386.19 ± 113.95	372.14 ± 107.03	0.568
Blood loss (ml)	534.6 ± 446.9	503.8 ± 409.5	0.78
Peritonectomy	18	205	0.283
Need for bowel anastomosis	8	37	0.002*
Visceral resection	5	43	0.309
**Early post-operative surgical complications**	12	40	0.001*
**Clavien-Dindo classification of early post-operative surgical complications**			
0	3	235	0.034*
I	1	39	
II	3	24	
III	11	24	
IV	6	10	
V	1	3	
**Duration of hospitalization (days)**	9.1 ± 6.17	8.3 ± 3.94	0.381

NACT: Neo-adjuvant chemotherapy; PCI: Peritoneal carcinomatosis index

**Table 4:** Multivariate regression analysis

	OR	95% CI		*p*-value
Age	0.46	0.29	0.66	0.89
Sex	0.8	0.54	1.31	0.56
**Comorbidities**				
Hypertension	0.55	0.28	1.66	0.75
Diabetes	0.26	0.06	0.58	0.64
Hypothyroidism	0.64	0.39	1.08	0.31
Obesity	1.4	0.85	1.69	0.005
**Post NACT status**	2.6	1.85	3.46	0.01
**PCI score**	1.15	0.94	1.52	0.09
Peritonectomy	1.26	0.91	1.95	0.52
Need for bowel anastomosis	1.13	1.02	1.21	0.03
Visceral resection	0.51	0.33	0.74	0.6
**Early post-operative surgical complications**	3.4	1.8	5.24	0.001
**Clavien-Dindo grade of post-operative complications**	2.9	1.52	3.66	0.001

NACT: Neo-adjuvant chemotherapy; PCI: Peritoneal carcinomatosis index

## Discussion

An incisional hernia is an unwanted but frequent complication that can occur after laparotomy for abdominal malignancies. Our single-center retrospective study revealed that the annual incidence of this complication after CRS and HIPEC was 3.5%, as assessed during a two-year follow-up period. This study also showed that patients who were obese, needed bowel anastomosis during surgery, experienced early post-operative surgical complications, mainly SSI, and who received neoadjuvant chemotherapy were at a significantly greater risk of developing an incisional hernia (Table 3).

Research by Struller et al. in Germany, focusing on patients who underwent CRS and HIPEC treatment, reported that 7% of the patients developed IH annually. They identified several factors that were linked to IH, including advanced age, cardiac comorbidities, mesothelioma, and pseudomyxoma peritonei [25]. Similarly, Ravn et al. from Denmark reported an annual incidence of 5.9% after a 60-month follow-up period in 152 patients who had undergone CRS and HIPEC. Sissel et al. reported a 5.9% annual incidence of IH after 60 months of follow-up in 152 patients. Those who developed IH were significantly older and had a greater rate of fascial dehiscence than those without IH [20]. Unlike our study, the average age of the study participants was more than 60 years, which could have affected the outcome as advanced age has been postulated as an independent risk factor for the development of IH after abdominal surgeries [23]. Moreover, colorectal and appendiceal carcinomas were the most common malignancies in both these studies, unlike this research, where ovarian carcinoma was the most common. However, the impact of primary malignancy on the incidence of IH after CRS and HIPEC is not well established.

In contrast to other studies, Ben-Yaacov et al. [21] reported a much greater incidence, with 29% of 201 patients developing IH within six months of undergoing CRS and HIPEC. Tuttle et al. also reported IH in 17% of patients at a median follow-up of 245 days. The duration of the follow-up period in both these researches was lesser than in the present study, which is crucial as longer follow-up may provide a better estimate of the long-term risk of developing IH after CRS and HIPEC.

Our study also revealed a significant association between IH and early post-operative surgical complications, the severity of complications (Clavien-Dindo classification), and the need for bowel anastomosis during surgery. This wide variation in the reported incidence of IH and associated factors in the literature is likely due to differences in patient demographics, varying definitions of IH, inconsistent follow-up durations, diverse surgical techniques, and discrepancies in the methods used to diagnose hernias.

The relationship between chemotherapy and IH is not well understood, and it is difficult to establish causality. Previous studies have reported that both pre-and post-operative chemotherapy can have detrimental effects on wound healing [26, 27]. In our study, we observed that the administration of NACT was significantly associated with the occurrence of IH. Campos et al. also reported that preoperative chemotherapy was a significant risk factor for IH after CRS and HIPEC for peritoneal surface malignancies [28]. According to Rettenmaier et al., the administration of chemotherapy during the perioperative period is a crucial factor in predicting an accelerated time for the development of IH [29]. In a study by Baucom et al. involving 491 patients who underwent abdominal malignancy surgery, the incidence of IH was 43% [8]. Claes et al. reported an IH rate of 35% after surgical resection of colorectal carcinomas [30]. After ovarian cancer surgery, the incidence of IH was 17.7% as estimated by Spencer et al. [31].

It is crucial to conduct regular CT scans to obtain a precise estimation of the incidence of IH. Studies that do not employ this method tend to underestimate the prevalence of IH. For example, a study on patients who underwent colorectal cancer surgery reported that the rate of IH detected on clinical examination was 17.4%. In contrast, the rate of detection by CT scans was 35% [30].

With the advancements in the medical field particularly in the field of oncology, the patients once considered palliative are offered complete cure with these complex CRS and HIPEC procedures. The most important aspect of following up with these patients post-surgery is to detect the recurrence of disease at the earliest and thereby offer timely intervention to these patients. The highlights of our study include the large sample size, enumeration of the possible factors affecting the incidence of IH, and the methods to prevent it. Our study also highlights the need for proper documentation of every post-operative outcome which may affect the incidence of IH, as they may be missed thereby affecting the quality of data available for analysis. We also hope that our article stimulates the establishment of a standard set of guidelines and surgical practices in reducing IH after CRS and HIPEC, as the literature on IH has been largely restricted to abdominal surgeries as a whole or just surgeries for abdominal malignancies rather than post CRS and HIPEC.

The most reliable way to diagnose IH is through CT imaging with the Valsalva maneuver [32, 33]. However, in our retrospective study, the primary focus of the CT scan was to identify tumor recurrence, thus the CT scan was conducted without the Valsalva maneuver in patients who did not clinically have incisional hernia before ordering a CT scan. Therefore, these scans may not be very sensitive for detecting IH in asymptomatic cases. Other limitations of this study include its retrospective design, which is inherently prone to selection and information bias. For instance, several technical details of the surgical procedure could not be obtained from the patient’s records retrospectively, such as the exact method of creating a bowel anastomosis, which could have varied over time and personal preference of surgeons. A follow-up period of two years may also be insufficient for estimating the long-term incidence of IH. As this study was conducted at a single centre, the incidence of each complication was insufficient to determine the strength of the association of individual complications with the incidence of IH. CRS and HIPEC procedures are done for a heterogeneously diverse group of diseases and organ systems with the indications for the procedure increasing day by day. Intrinsic variations in tumor biology and patterns of spread may decide the extent of CRS and HIPEC thereby affecting the incidence of IH. Hence large volume prospective multicentric studies may be required to validate our findings and to provide more insight on the subset of patients more prone to IH.

Considering the high chances of IH following CRS and HIPEC, it is suggested to adopt certain measures to decrease the incidence. The measures include optimizing the surgical technique with minimal tissue trauma and ensuring a meticulous closure of the abdominal wall. This can be achieved through reinforcement with mesh, with appropriate suture materials and careful tissue handling of the wound. The patient characteristics should be taken into account like BMI, comorbidities and nutritional status and provide them with a tailoring surgical approach and perioperative care. Early postoperative mobilization is recommended with strong rehabilitation programs for strengthening abdominal muscles [1–5]. 

## Conclusion

The incidence of IH after CRS and HIPEC in our study was lower than the incidence ranges described in previous studies. Obesity, early post-operative complications, intraoperative bowel anastomosis, and post-NACT status may increase the likelihood of developing IH. The findings of this study can help in improving the selection of patients for CRS and HIPEC procedures. It can also aid in providing pre-operative counseling to individuals regarding the development of IH and identifying those who are at risk of developing it. Additionally, this study will guide future researchers in designing and conducting prospective randomized trials to obtain better-quality evidence. The only investment made was a little extra attention given to the patient.

## Data Availability

No datasets were generated or analysed during the current study.

## Declarations

### Study design

Retrospective analysis of prospectively maintained database.

## Abbreviations

IH: incisional hernia
CRS: cytoreductive surgery
HIPEC: hyperthermic intraperitoneal chemotherapy
CC: completion of cytoreduction
CT: computed tomography
PCI: peritoneal carcinomatosis index
PDS: polydioxanone
NACT: neoadjuvant chemotherapy
